# All-cause mortality rate in China: do residents in economically developed regions have better health?

**DOI:** 10.1186/s12939-020-1128-6

**Published:** 2020-01-21

**Authors:** Xuexin Yu, Wei Zhang

**Affiliations:** West China Biomedical Big Data Center, West China Hospital, Sichuan University, 37 Guo Xue Alley, Chengdu, 610040 Sichuan China

**Keywords:** All-cause mortality rate, Population health, China, Regional disparities, Urban-suburban-rural disparities

## Abstract

**Background:**

Urban-rural disparities have been extensively investigated, while most investigators overlooked urban-suburban-rural variations in population health. Although regional disparities in East-West China have been largely discussed, limited attention has been directed to the interaction between regional differences and urban-suburban-rural disparities. This study aims to analyze urban-suburban-rural variations in all-cause mortality rates across four geographic regions in China.

**Methods:**

Data came from China’s National Census Survey and public statistical yearbooks in 2000 and 2010. Urban districts, county-level cities, and counties were respectively defined as urban, suburban, and rural areas. We obtained 2322 areas, including 2148 areas with two observations and 174 areas with only one observation. Data visualization was performed to depict geographic variations and changes in all-cause mortality rates. Five hierarchical linear regression analyses with generalized estimating equations (GEE) were employed to analyze variations in all-cause mortality rates over time. Demographic and socioeconomic attributes were introduced as covariates.

**Results:**

Despite an overall decline in all-cause mortality rate, rural residents generally achieved worse health than urban and suburban counterparts. In contrast, urban-suburban disparities could be fully explained by demographic and socioeconomic differences. In addition, Northeastern and Central residents achieved better health than Eastern and Western residents. Last, there existed urban/suburban-rural disparities in all regions, except Northeastern, where urban/suburban-rural disparities were eliminated after controlling for socioeconomic and demographic attributes.

**Conclusion:**

Even though suburban and rural areas were often merged, there exist urban/suburban-rural disparities in population health. Furthermore, urban/suburban-rural disparities vary across regions.

## Background

As a key issue of health equity, urban-rural disparities have been extensively discussed [1, 2], while prior observations have directed limited attention to urban-suburban-rural disparities. Even though Fogelholm et al. noted that there existed urban-suburban-rural differences in the health of the older population in Finland [3], limited research has focused on urban-suburban-rural disparities in mainland China, where suburban areas were often merged into rural areas [4, 5].

A fair number of individual-level observations in China have noted geographic variations in residents’ health with measures including comorbidity of chronic disease [6], quality of life [7], and self-rated health [8]. In contrast, regional-level studies in China tended to employ a provincial-level analysis. For example, Zhou et al. noted that residents in metropolitan cities in Eastern China reported higher life expectancy and longer quality-adjusted life years [9]. The provincial-level analysis, however, may not fully capture county-level variations and thereby failed to provide practical policy recommendations [9].

Most importantly, limited observations have investigated the interaction between geographic regions and urban-suburban-rural settings. Even though Sun et al. noted that disparities among rural areas were more profound than those among urban areas in China, they did not examine urban-suburban-rural disparities across regions [7]. As the progress of urbanization [10], transportation system [11, 12], landform [11], distribution of health care resources [13], and health care utilization [5, 13, 14] differed, urban-suburban-rural disparities may not be consistent across regions.

### Hypotheses

This study aimed to analyze variations in all-cause mortality rates across urban, suburban, and rural China in 2000 and 2010. To offer guidance for data analysis, we offered two hypotheses here.

First, we hypothesized that urban-suburban-rural disparities in all-cause mortality rates existed even after controlling for demographic and socioeconomic attributes (H1) since urban residents generally have better living conditions, easily accessible health care as well as higher health literacy, which may not be fully captured in demographic and socioeconomic differences [2, 15].

Second, we hypothesized that the urban-suburban-rural disparities might vary across regions (H2), as heterogeneity exists not only in economic development but also in the transportation system [16], the distribution of health care resources [13], and epidemiologic characteristics [9] among four geographic regions. On the one hand, economically developed metropolitan cities in Eastern and Central China may centralize health care resources, which may or may not be conducive to residents’ health in neighboring suburban and rural areas with insufficient health care resources. On the other hand, the broad plateau landform in rural areas of Western China may hinder residents from seeking health services in neighboring urban areas [17]; thereby, rural residents in Western China may achieve worse health relative to their counterparts in other three regions.

## Materials and methods

### Data sources

The present study employed a longitudinal study design with data from 2000 and 2010. Data were derived from China’s National Census Survey (county data) in 2000 and 2010 [18, 19], China City Statistical Yearbooks in 2001 and 2011 [20, 21], and China County Statistic Yearbooks in 2001 and 2011 [22, 23]. Demographic data (i.e., the number of residents, the population aged under 5, the population aged over 65, female-to-male ratio, minority, migrant, populations with a high school education or above) were derived from National Census Survey. Data about the land area of counties, county-level cities, and urban districts were derived from China County Statistic Yearbook and China City Statistical Yearbook, respectively.

### Study units

In this study, we included data of urban, suburban, and rural areas in mainland China, while we did not include data of Hongkong, Macau, and Taiwan due to data limitation. In our definition, urban districts in the same prefectural or municipal city were merged and treated as an individual urban unit. In addition, we respectively treated each county-level city and rural county as an individual suburban and rural unit, which was consistent with the National Census Survey and China City Statistic Yearbook [18, 20].

In the 2000 wave, after excluding 121 units (5.12%) with missing values, we obtained 2242 study units, which included 1607 rural units (counties), 369 suburban units (county-level cities), and 266 urban units. In the 2010 wave, we excluded 53 units (2.32%) with missing data, and obtained 2228 study units, including 1600 rural units, 349 suburban units, and 279 urban units. Overall, we obtained data of 2322 study units, including 2148 study units with two observations and 174 study units with only one observation.

### Measures

The all-cause mortality rate was measured as the dependent variable. As local demographic characteristics often affect population health, we included the population aged under 5 (%), the population aged over 65 (%), female-to-male ratio (%), population density (the number of residents divided by land area, /km^2^), and minority (%) as independent variables [8, 24, 25]. In addition, populations with a high school education or above (%) and migrant (%) were introduced as covariates since they reflect socioeconomic status, which may lead to health disparities [8, 26]. As a critical issue in this study, urban–suburban-rural settings were introduced to reflect the administrative level and location of the study unit according to the National Census Survey and the China City Statistic Yearbook [18, 20]. In addition, conforming to the bulletin of the National Bureau Statistics of China, regional variations were captured as three dummies, i.e., Eastern, Central, and Northeastern China, with Western China as the reference [27].

### Data analyses

**Fig. 1:**
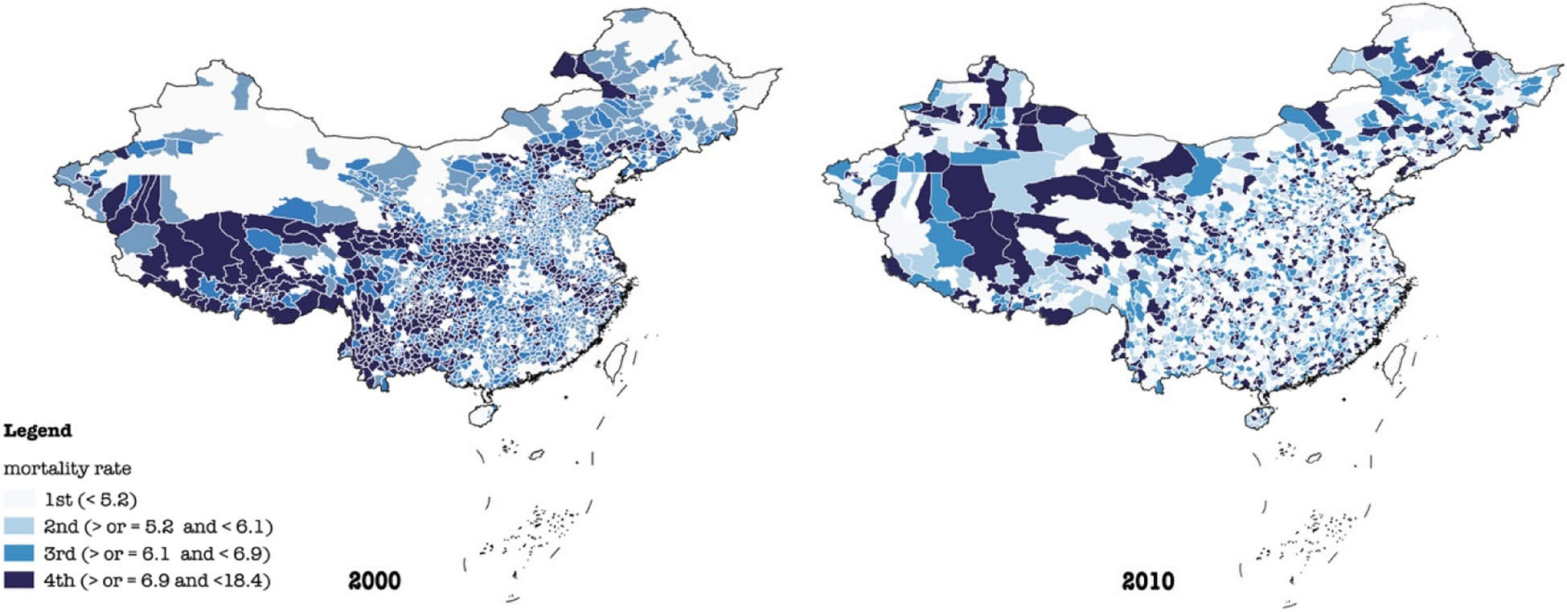
All-cause mortality rate in China in 2000 and 2010

**Fig. 2:**
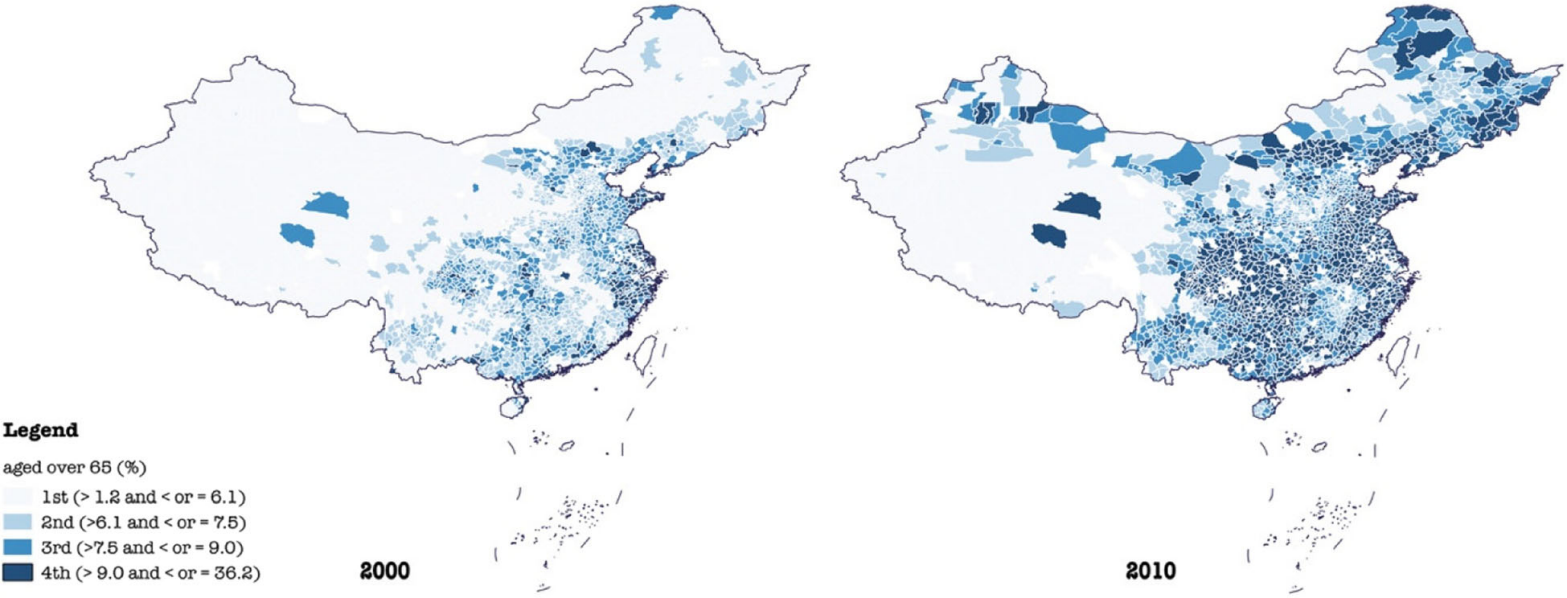
Distribution of the population aged over 65 (%) in China in 2000 and 2010

**Fig. 3:**
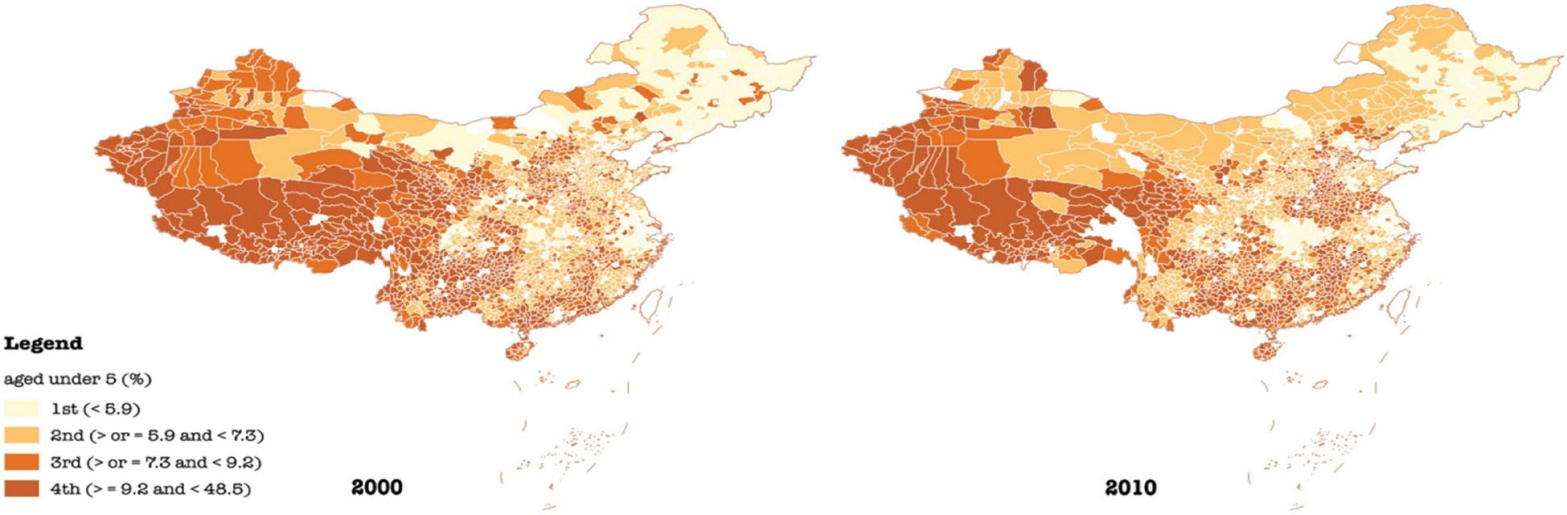
Distribution of the population aged under 5 (%) in China in 2000 and 2010

To depict geographic variations and changes in the all-cause mortality rate (%), the population aged over 65 (%), and the population aged under 5 (%) in China in 2000 and 2010, we generated six geographic maps in Figs. 1, 2, and 3. To gauge differences in baseline characteristics in urban-suburban-rural areas as well as four regions, we presented mean values of each variable in Table 1. As the skewness and kurtosis of dependent variables were moderate and acceptable, five hierarchical linear regression analyses with generalized estimating equations (GEE) were performed. With reference to urban-suburban-rural disparities across regions, 11 dummies reflecting the interaction of four geographic regions and urban-suburban-rural settings were introduced in model 5 with rural areas in Western China served as the reference. Variance inflation factors (VIF) were calculated to examine collinearity among independent variables, results of which suggest slight multicollinearity (VIF < 4).

**Table 1:** Baseline characteristics (mean values)

Region	Variable	2000			2010		
		Rural	Suburban	Urban	Rural	Suburban	Urban
Central		*N* = 408	*N* = 88	*N* = 78	*N* = 410	*N* = 85	*N* = 79
	all-cause mortality rate (%)	6.38	5.91	4.67	5.99	5.37	4.48
	High school education or above (%)	8.19	11.05	21.90	16.84	22.13	37.06
	migrant (%)	4.61	6.99	19.1	8.32	10.73	24.91
	aged under 5 (%)	8.74	7.26	7.83	8.24	7.47	6.32
	aged over 65 (%)	5.84	6.30	6.67	8.92	9.01	8.19
	population density (/km2)	115.54	175.01	1010.45	318.74	456.01	1319.82
	female-to-male ratio (%)	94.34	94.99	97.51	94.78	96.02	96.69
	minority (%)	12.46	15.10	6.64	5.58	2.64	2.09
Northeastern		*N* = 95	*N* = 56	*N* = 34	*N* = 92	*N* = 55	*N* = 34
	all-cause mortality rate (%)	5.42	5.71	4.88	5.76	5.80	5.53
	high school education or above (%)	10.67	13.09	24.77	16.12	19.67	36.88
	migrant (%)	7.10	9.43	19.74	9.65	12.79	24.00
	aged under 5 (%)	6.60	6.21	5.24	5.08	4.63	3.78
	aged over 65 (%)	5.84	6.30	6.67	8.23	9.14	9.79
	population density (/km2)	115.54	175.01	1010.45	111.16	173.77	845.78
	female-to-male ratio (%)	94.34	94.99	97.51	96.11	96.54	98.75
	minority (%)	12.46	15.1	6.64	11.66	13.75	6.06
Western		*N* = 763	*N* = 80	*N* = 68	*N* = 763	*N* = 71	*N* = 84
	all-cause mortality rate (%)	6.82	5.41	5.03	6.26	4.73	4.67
	high school education or above (%)	6.47	12.91	17.83	14.22	22.99	29.45
	migrant (%)	6.04	15.07	17.00	10.28	21.06	27.27
	aged under 5 (%)	10.54	8.91	8.34	7.97	6.56	6.29
	aged over 65 (%)	5.95	5.88	6.18	8.14	8.47	8.41
	population density (/km2)	139.18	316.92	749.35	129.09	337.16	632.60
	female-to-male ratio (%)	92.46	92.71	93.02	93.30	94.13	95.07
	minority (%)	41.83	26.60	11.81	42.04	27.32	14.28
Eastern		*N* = 341	*N* = 145	*N* = 86	*N* = 335	*N* = 138	*N* = 82
	all-cause mortality rate (%)	6.39	6.36	4.70	6.79	5.86	4.73
	high school education or above (%)	8.64	10.54	21.95	16.71	20.54	35.44
	migrant (%)	6.05	10.49	27.19	10.87	18.16	33.60
	aged under 5 (%)	7.82	8.03	6.76	8.08	6.56	5.81
	aged over 65 (%)	7.90	8.44	6.92	9.63	9.98	8.22
	population density (/km2)	410.53	584.28	1710.36	415.57	639.31	1716.8
	female-to-male ratio (%)	95.35	96.87	95.90	96.32	96.61	95.71
	minority (%)	3.73	1.69	2.05	4.01	2.10	1.90
Overall		*N* = 1607	*N* = 369	*N* = 266	*N* = 1600	*N* = 349	*N* = 279
	all-cause mortality rate (%)	6.53	5.94	4.80	6.27	5.50	4.74
	high school education or above (%)	7.62	11.56	21.24	15.52	21.29	34.27
	migrant (%)	5.74	10.49	21.26	9.87	16.10	28.06
	aged under 5 (%)	9.27	7.76	7.28	7.90	6.48	5.85
	aged over 65 (%)	6.63	7.18	6.63	8.66	9.30	8.46
	population density (/km2)	241.2	434.86	1282.9	236.64	459.83	1171.82
	female-to-male ratio (%)	93.15	94.85	94.97	94.48	95.95	96.17
	minority (%)	22.77	9.37	5.20	22.98	9.20	6.19

Data visualization was performed with QGIS 2.18. Statistical analyses were performed with Stata/SE 15.0 (StataCorp, TX, USA). A two-tailed *P* value of less than 0.05 was considered statistically significant.

## Results

### Descriptive analyses

Results from descriptive analyses suggest that the overall all-cause mortality rate declined in China (Table 1 & Fig. 1). In 2000, all-cause mortality rates ranged from 4.80% in urban areas, 5.94% in suburbs, to 6.53% in rural areas, all of which decreased in 2010 (4.74% in urban areas, 5.50% in suburban areas, and 6.27% in rural areas).

The changes, however, differed across regions. First, there existed a similar decrease in all-cause mortality rates of Central and Western China. Specifically, all-cause mortality rates in urban, suburban, and rural areas in these two regions declined from 2000 to 2010 (Table 1). In contrast, all areas in Northeastern and urban as well as rural areas in Eastern China witnessed an increase in all-cause mortality rate from 2000 to 2010.

Likewise, comparisons within urban, suburban, and rural areas appeared to differ across regions (Table 1). First, urban areas in Central China had the lowest mortality rate in both 2000 and 2010 (4.67% in 2000 and 4.48% in 2010), relative to their counterparts in Northeastern (4.88% in 2000 and 5.53% in 2010), Western (5.03% in 2000 and 4.67% in 2010), and Eastern China (4.70% in 2000 and 4.73% in 2010). While among suburbs, Western China had the lowest all-cause mortality rate (5.41% in 2000 and 4.73% in 2010) compared to Northeastern (5.71% in 2000 and 5.80% in 2010), Central (5.91% in 2000 and 5.37% in 2010), and Eastern China (6.36% in 2000 and 5.86% in 2010). Finally, it was rural areas in Northeastern China (5.42% in 2000 and 5.76% in 2010) that had the lowest mortality rate relative to their counterparts in Central (6.38% in 2000 and 5.99% in 2010), Eastern (6.39% in 2000 and 6.79% in 2010), and Western China (6.82% in 2000 and 6.26% in 2010).

In addition, socioeconomic status and demographic attributes differed across not only urban-suburban-rural areas but also geographic regions (Table 1, Fig. 2 & Fig. 3). In terms of the proportion of populations with a high school education or above, urban areas in Western China (17.83% in 2000 and 29.45% in 2010) were outweighed by their counterparts in Central (21.90% in 2000 and 37.06% in 2010), Northeastern (24.77% in 2000 and 36.88% in 2010), and Eastern China (21.95% in 2000 and 35.44% in 2010). Rural areas in Western China (6.47% in 2000 and 14.22% in 2010) presented similar weakness compared with Northeastern (10.67% in 2000 and 16.12% in 2010), Central (8.19% in 2000 and 16.84% in 2010), and Eastern China (8.64% in 2000 and 16.71% in 2010) as well. It was, however, suburbs in Eastern China (10.54% in 2000 and 20.54% in 2010) that had the lowest education status relative to suburbs in Western (12.91% in 2000 and 22.99% in 2010), Central (11.05% in 2000 and 22.13% in 2010), and Northeastern China (13.09% in 2000 and 19.76% in 2010).

Furthermore, despite an overall increase in the proportion of the population aged over 65, suburban areas generally had a higher proportion of older population (7.18% in 2000 and 9.30% in 2010) than urban (6.63% in 2000 and 8.46% in 2010) and rural areas (6.63% in 2000 and 8.66% in 2010). While the proportion of the population aged under 5 declined from 2000 to 2010 with rural areas having the highest proportion of young children (9.27% in 2000 and 7.90% in 2010) compared with suburbs (7.76% in 2000 and 6.48% in 2010) and urban areas (7.28% in 2000 and 5.85% in 2010).

#### GEE analyses

##### Regional variations

Results from model 1 (Table 2) suggest that all-cause mortality rates in Northeastern and Central China were 0.70 and 0.38 lower than that of Western China, respectively. Results were robust even after controlling for demographic and socioeconomic differences in model 4 (Northeastern vs. Western, β = − 0.27, *P* < .001; Central vs. Western β = − 0.26, *P* < .001). Differences in all-cause mortality rates between Eastern and Western China, however, were not statistically significant (β = 0.07, *P* > .05). Results here appear to suggest that residents in Northeastern and Central China had better health than those in Eastern and Western China.

**Table 2:** GEE analyses of all-cause mortality rate (%) in 2000 and 2010

independent variables	model 1		model 2		model 3		model 4		model 5	
	β	95% CI	β	95% CI	β	95% CI	β	95% CI	β	95% CI
year 2010 (vs. 2000)	−0.27^***^	(−0.33, −0.21)	−0.27^***^	(−0.32, −0.21)	−0.21^***^	(−0.30, −0.13)	−0.37^***^	(−0.46, −0.28)	− 0.37^***^	(− 0.46, − 0.28)
Eastern (vs. Western)	−0.08	(− 0.20, 0.05)					−0.07	(−0.18, 0.04)		
Northeastern (vs. Western)	− 0.70^***^	(− 0.89, − 0.51)					−0.27^***^	(− 0.41, − 0.12)		
Central (vs. Western)	−0.38^***^	(− 0.51, − 0.26)					− 0.26^***^	(− 0.37, − 0.16)		
urban (vs. rural)			−1.62^***^	(−1.76, −1.49)			−0.59^***^	(− 0.75, − 0.43)		
suburban (vs. rural)			−0.68^***^	(−0.80, − 0.55)			− 0.47^***^	(− 0.58, − 0.37)		
Interaction										
Western x rural									Ref.	
Eastern x urban									−0.77^***^	(− 1.02, −0.52)
Eastern x suburban									−0.58^***^	(−0.75, − 0.41)
Eastern x rural									−0.18^**^	(−0.30, − 0.05)
Northeastern x urban									−0.47^**^	(−0.80, − 0.14)
Northeastern x suburban									−0.55^***^	(−0.79, − 0.31)
Northeastern x rural									−0.69^***^	(−0.88, − 0.50)
Central x urban									−0.93^***^	(−1.17, − 0.68)
Central x suburban									−0.69^***^	(−0.89, − 0.49^f^)
Central x rural									−0.38^***^	(−0.49^g^, − 0.26)
Western x urban									−0.90^***^	(−1.12, − 0.68)
Western x suburban									−0.99^***^	(−1.20, − 0.79)
high school education or above (%)					−0.06^***^	(−0.07, − 0.05)	−0.04^***^	(− 0.05, − 0.04)	−0.04^***^	(− 0.05, − 0.04)
migrant (%)					−0.01^***^	(−0.02, − 0.01)	−0.01^***^	(− 0.02, − 0.01)	−0.01^***^	(− 0.01, 0.00)
aged under 5 (%)					0.02^***^	(0.01, 0.03)	0.01^***^	(0.01, 0.02)	0.01^***^	(0.00, 0.02)
aged over 65 (%)					0.27^***^	(0.25, 0.29)	0.27^***^	(0.25, 0.29)	0.26^***^	(0.24, 0.28)
population density					−0.00 ^a^	(0.00, 0.00)	−0.00 ^b^	(0.00, 0.00)	−0.00 ^c^	(0.00, 0.00)
female-to-male ratio (%)					0.01	(0.00, 0.02)	0.01^***^	(0.01, 0.02)	0.02^***^	(0.01, 0.02)
minority (%)					0.01^***^	(0.00, 0.01)	0.00^*** d^	(0.00, 0.01)	0.00^*** e^	(0.00, 0.01)

Note: *** *P* < .001; ** *P* < .01; * *P* < .05

^a^0.0000659; ^b^0.000015; ^c^0.0000262; ^d^0.004; ^e^0.004; ^f^0.4912; ^g^0.4902

##### Urban-suburban-rural settings

With reference to urban-suburban-rural disparities, results from model 2 demonstrate that urban (β = − 1.62, *P* < .001) and suburban (β = − 0.68, *P* < .001) residents achieved lower all-cause mortality rates compared with rural residents (Table 2), which were consistent after controlling for demographic and socioeconomic attributes in model 4 (urban vs. rural, β = − 0.59, *P* < .001, 95% CI, [− 0.75, − 0.43]; suburban vs. rural, β = − 0.47, *P* < .001, 95% CI, [− 0.58, − 0.37]). Results here suggest that urban/suburban-rural disparities existed even when population and socioeconomic characteristics were introduced, while urban-suburban disparities were eliminated after controlling for population and socioeconomic characteristics, since the 95% CI of the coefficient of suburban areas overlapped with that of urban areas in model 4. We have changed the reference group to suburban areas to undertake supplemental analysis, results of which support the interpretation in terms of the overlap (results not presented here). Results here partially support our first hypothesis (H1).

##### Urban-suburban-rural settings & geographic regions

Results from model 5 further indicate that urban/suburban-rural variations in all-cause mortality rates varied across regions (H2). First, urban/suburban-rural disparities existed in Eastern, Central, and Western China, since the 95% CI of the coefficient of rural areas did not overlap with that of urban areas as well as suburbs in these three regions, whereas the coefficient’s 95% CI of suburbs and that of urban areas overlapped. We have respectively changed the reference group to rural areas in Eastern, Northeastern, and Central China to undertake supplemental analyses, results of which support the interpretation in the present and following paragraph (results not presented here).

Urban/suburban-rural disparities, however, did not exist in Northeastern region, since the 95% CI of urban, suburban, and rural areas in Northeastern China overlapped, which suggests no disparities existed in urban, suburban, and rural areas in Northeastern (rural areas in Northeastern China vs. rural areas in Western China, β = − 0.69, 95%CI, [− 0.88, − 0.50]; suburban areas in Northeastern China vs. rural areas in Western China, β = − 0.55, 95%CI, [− 0.79, − 0.31]; urban areas in Northeastern China vs. rural areas in Western China, β = − 0.47, 95%CI, [− 0.80, − 0.14]).

In addition, rural areas in Western China had the highest all-cause mortality rate after controlling for all other covariates in model 5. Moreover, all-cause mortality rates in rural areas of Eastern China (β = − 0.18, *P* < .01) and Central China (β = − 0.38, *P* < .001) were slightly lower than those of rural areas in Western China.

##### Social determinants

Furthermore, our results suggested that residents in a place with higher education attainment (β = − 0.04, *P* < .001) and a higher proportion of the migrant population (β = − 0.01, *P* < .01) appeared to achieve lower mortality even after controlling for all other covariates in model 3. Moreover, a 1% increase in the proportion of the population aged under five and the population aged over 65 may respectively lead to 0.01 and 0.27% increase in all-cause mortality rate after controlling for all other covariates in model 3. Last, a higher proportion of female (β = 0.01, *P* < .001) and minority population (β = 0.004, *P* < .001) appeared to be positively associated with all-cause mortality rate even when covariates were introduced in model 3 (Table 2).

## Discussion

This study highlights the urban-suburban-rural variations in China, where suburban areas were often mixed with rural areas [4]. There is a rich empirical evidence on urban-rural disparities [1, 4]. Nevertheless, most observations have overlooked variations across urban, suburban, and rural areas. Even though a fair number of prior studies have investigated geographic variations in China’s population health, they provided insufficient information with differences after adjusting for socioeconomic and demographic attributes [13]. It is essential to bridge the knowledge gap since it may play a key role in targeting underserved areas for health policy practice and health promotion programs [1, 4, 28–30]. Most importantly, this study would contribute to the literature evidence on population health in rural China since most studies in China were based on city-level or province-level data, which may not fully capture health variations among rural counties [9].

This study has three interesting findings. First, rural areas in Western China were major sources of urban-suburban-rural disparities, followed by rural areas in Central and Eastern China. Results here were in line with a number of prior studies. For example, Sun et al. suggested that individuals in rural areas of Central and Eastern China achieved a better quality of life than those of Western China [7]. Likewise, Wang et al. noted that rural counties in Western China had higher infant mortality rates relative to their counterparts in Central and Eastern China [28]. It is not surprising that residents achieved worse health in rural areas of Western China due to the transportation/health-unfriendly plateau landform [17], the endemic infectious diseases [9], and uncultured infrastructure [10].

However, Sun et al. and Wang et al. merged Northeastern China with Eastern China [7, 28], which may underestimate the East-West disparities among rural areas, since our results suggested that residents in rural areas of Northeastern achieved even better health than their counterparts of Eastern China. Results from several prior studies could partially support the health advantage in Northeastern China. For example, Gu et al. found that the oldest-old in Northeastern China had the highest probability of self-reporting good health relative to those in Eastern and Western China [31]. The reasons for the variation, however, are not clear and may be attributed to multiple factors. One possibility is that urban areas in Central and Eastern China assembled highly-dense health care resources, which hindered the health care distribution in neighboring rural regions. This possibility is supported the fact over 50% top-tier hospitals were located in urban cities in Eastern and Central China [32]. The other possibility is that rural residents in Eastern, Central, and Western China may have greater life pressure due to the rapid urbanization relative to rural residents in Northeastern China, where the increase in socioeconomic attributes (i.e., the proportion of populations with a high school education or above) did not keep up with that in other three regions (Table 1). This possibility is further supported by two prior studies, in which results suggested that rural residents in Northeastern China had the lowest suicide rate [33] and the lowest prevalence of hypertension relative to residents in other regions [34].

Second, even though suburban and rural areas were often merged and collectively treated as rural areas in prior studies [4, 35], suburban-rural disparities existed in Eastern, Central, and Western China. In addition, population health in the suburbs was actually similar to that in urban areas after controlling for population and socioeconomic attributes. Results here were consistent with those from a prior individual-level study, in which personal attributes could fully explain suburban-urban disparities, but urban/suburban-rural variations remained even after controlling for demographic characteristics, education attainment, and health behaviors [36]. In this regard, the cost-effectiveness of health-promoting program and practice may be diminished according to the prior classification, since limited resources may be distributed to suburbs which were mixed in rural settings.

Third, we found that urban/suburban-rural disparities varied across regions. More specifically, rural areas were generally dominated by their neighboring urban areas except those in Northeastern China. On the one hand, results here parallel to the urban-rural gap in prior studies, in which results suggest that residents in rural areas had a higher probability of poor self-rated health [37] and had higher suicide rates [33]. Even though rural areas in China have been undergoing rapid social development [38], urban-rural disparities remained in residents’ access to health care [2], which may not be significantly improved in a short period. Therefore, urban-rural disparities would still be a challenging issue in China, albeit the rapid economic growth and universal health insurance coverage [39].

On the other hand, our results suggest that urban-rural disparities did not exist in all geographic regions in China, and urban-rural disparities in Northeastern China appeared to be fully explained by demographic and socioeconomic attributes. However, our results here differ from those in previous observations. For example, Wang et al. noted that rural residents in Northeastern China were more likely to have several types of chronic diseases even after controlling for individual-level covariates relative to urban residents in the same region [40], while the result may not directly confirm the urban-rural gap in residents’ mortality rate. Further research may be necessary in order to bridge the knowledge gap in Northeastern China in terms of urban-rural variations in population health.

Our results draw several policy implications. First, urban-suburban disparities could be eliminated by boosting socioeconomic development in all regions, while urban/suburban-rural disparities would not be entirely smoothed by merely promoting social development in Eastern, Western, and Central China. For these three regions, policymakers may consider proposing comprehensive strategies to address urban-rural disparities, such as promoting rural residents’ health literacy [41] and boosting village-level social engagement [42], which is also important for countries that are confronted with similar health disparities. Furthermore, rural areas in Eastern, Central, and Western China were major sources of urban/suburban-rural disparities. For the cost-effectiveness of health-promoting programs, removing suburban areas from rural areas, which were often mixed before [4], and targeting rural areas in Eastern, Central, and Western China hold promise. Last, it would be helpful to examine the interaction between geographic regions and urban-rural settings in countries, such as the United States, where health disparities exist not only in urban-rural areas but also across West, Midwest, Southeast, and Northeast states [43].

This study presents an overall improvement compared with prior studies. First, prior observations analyzing geographic variations in health tended to employ a provincial-level analysis and a cross-sectional study design, while this study entailed multivariate analysis to depict health variations across over 2200 areas over time. In addition, instead of urban-rural variations, we further investigated urban-suburban-rural disparities in population health and conceptualized all-cause mortality rates in the context of socioeconomic development. Additionally, we analyzed urban-suburban-rural disparities across regions, which targeted the most underserved area and provided more practical and region-specific policy implications. Even though geographic and urban-rural variations may differ across countries, and one should be cautious to generalize our results to other countries, research concepts and methodology of the present study may provide an exemplar for future research in terms of conceptualizing health disparities.

Despite the strength, this study has several limitations. First, we employed all-cause mortality rates as health outcomes, which may not be an ideal measure, even though multiple covariates have been introduced. Ideally, analysis in variations of aged/sex-adjusted mortality rates [9], infant mortality rates [4], premature mortality rates [44], and quality-adjusted life years [9] should be included. In addition, we employed a relatively outdated data set due to data limitation, which may undermine the representativeness of China’s current conditions.

## Data Availability

All data came from public domain and could be accessed from the link below (CNKI readers limited). 1) National Census survey: https://125.70.226.87:543/web/1/http/2/tongji.cnki.net /kns55/Navi/HomePage.aspx?id=N2007080127&name=YWQUX&floor=1. [in Chinese]. 2) China City Statistical Yearbook: https://125.70.226.87:543/web/1/http/2/tongji. cnki.net/kns55/Navi/HomePage.aspx?id=N2017060038&name=YZGCA&floor=1. [in Chinese]. 3) China County Statistic Yearbook: http://data.cnki.net/Yearbook/Single/N2012020067. [in Chinese].

## Abbreviations

CI: Confidence interval
GEE: Generalized estimating equations
Ref: Reference
vs: Versus
